# Extensive Survey and Analysis of Factors Associated with Presence of Antibodies to Orthoebolaviruses in Bats from West and Central Africa

**DOI:** 10.3390/v15091927

**Published:** 2023-09-15

**Authors:** Martine Peeters, Maëliss Champagne, Innocent Ndong Bass, Souana Goumou, Simon-Pierre Ndimbo Kumugo, Audrey Lacroix, Amandine Esteban, Dowbiss Meta Djomsi, Abdoul Karim Soumah, Placide Mbala Kingebeni, Flaubert Auguste Mba Djonzo, Guy Lempu, Guillaume Thaurignac, Eitel Mpoudi Ngole, Charles Kouanfack, Daniel Mukadi Bamuleka, Jacques Likofata, Jean-Jacques Muyembe Tamfum, Helene De Nys, Julien Capelle, Abdoulaye Toure, Eric Delaporte, Alpha Kabinet Keita, Steve Ahuka Mundeke, Ahidjo Ayouba

**Affiliations:** 1 TransVIHMI, University of Montpellier, Institut National de la Santé et de la Recherche Médicale (INSERM), Institut de Recherche pour le Développement (IRD), 34394 Montpellier, France; maeliss.champagne@ird.fr (M.C.); audrey.lacroix@ird.fr (A.L.); amandine.esteban@ird.fr (A.E.); guillaume.thaurignac@ird.fr (G.T.); eric.delaporte@ird.fr (E.D.); 2 Centre de Recherche sur les Maladies Emergentes et Réémergentes (CREMER), Yaounde P.O. Box 1857, Cameroon; ssabgnodn@yahoo.com (I.N.B.); medjodow@yahoo.fr (D.M.D.); mbaflaubertz75@gmail.com (F.A.M.D.); charleskouanfack@yahoo.fr (C.K.); 3 Centre de Recherche et de Formation en Infectiologie de Guinée (CERFIG), Université Gamal Abdel Nasser de Conakry, Conakry BP6629, Guinea; souana.goumou@cerfig.org (S.G.); abdoul.soumah@cerfig.org (A.K.S.); abdoulaye.toure@ird.fr (A.T.); alpha-kabinet.keita@ird.fr (A.K.K.); 4 National Institute of Biomedical Research (INRB), Kinshasa P.O. Box 1197, Democratic Republic of the Congo; simonp_ndimbok@yahoo.fr (S.-P.N.K.); mbalaplacide@gmail.com (P.M.K.); kajosmpiana@gmail.com (G.L.); drmukadi@gmail.com (D.M.B.); jjmuyembet@gmail.com (J.-J.M.T.); amstev04@yahoo.fr (S.A.M.); 5 Service de Microbiologie, Cliniques Universitaires de Kinshasa, Kinshasa P.O. Box 1197, Democratic Republic of the Congo; 6 Laboratoire Provincial de Mbandaka, Equateur, Democratic Republic of the Congo; jacqueslikofata@gmail.com; 7 Astre, CIRAD, INRAE, University of Montpellier, 34398 Montpellier, France; helene.de_nys@cirad.fr (H.D.N.); julien.capelle@cirad.fr (J.C.); 8 Astre, CIRAD, 6 Lanark Road, Harare, Zimbabwe

**Keywords:** Ebola, Africa, bats, virus, antibody

## Abstract

The seroprevalence to orthoebolaviruses was studied in 9594 bats (5972 frugivorous and 3622 insectivorous) from Cameroon, the Democratic Republic of Congo (DRC) and Guinea, with a Luminex-based serological assay including recombinant antigens of four *orthoebolavirus* species. Seroprevalence is expressed as a range according to different cut-off calculations. Between 6.1% and 18.9% bat samples reacted with at least one orthoebolavirus antigen; the highest reactivity was seen with Glycoprotein (GP) antigens. Seroprevalence varied per species and was higher in frugivorous than insectivorous bats; 9.1–27.5% versus 1.3–4.6%, respectively. Seroprevalence in male (13.5%) and female (14.4%) bats was only slightly different and was higher in adults (14.9%) versus juveniles (9.4%) (*p* < 0.001). Moreover, seroprevalence was highest in subadults (45.4%) when compared to mature adults (19.2%), (*p* < 0.001). Our data suggest orthoebolavirus circulation is highest in young bats. More long-term studies are needed to identify birthing pulses for the different bat species in diverse geographic regions and to increase the chances of detecting viral RNA in order to document the genetic diversity of filoviruses in bats and their pathogenic potential for humans. Frugivorous bats seem more likely to be reservoirs of orthoebolaviruses, but the role of insectivorous bats has also to be further examined.

## 1. Introduction

Since the first recognized *Ebolavirus* disease (EVD) outbreaks in 1976 in the Democratic Republic of Congo (DRC) and Sudan, more than 35 outbreaks have been reported in Africa [1]. However, their frequency and impact has increased over the last decades as illustrated by the recent outbreaks in West Africa (December 2013 to March 2016) and in eastern DRC (August 2018 to June 2020) where thousands of individuals became infected over large geographic areas [1]. This is in contrast with the majority of previous EVD outbreaks that were in remote areas and remained geographically restricted. Today, all outbreaks have occurred in Africa and are caused by different viruses from the *Orthoebolavirus* genus. To remove the ambiguity between the genus *Ebolavirus* and the virus name Ebola virus (EBOV) for the previously called Ebola Zaire virus, the genus has been renamed *Orthoebolavirus* by the *Filoviridae* study group of the International Committee on Taxonomy of Viruses (ICTV) [2]. The Ebola virus (EBOV) is responsible for the majority of outbreaks in Central Africa and the large West African epidemic. Sudan virus (SUDV) and Bundibugyo virus (BDBV) have been reported in outbreaks in eastern Africa (Sudan, Uganda, eastern DRC) and Tai Forest virus (TAFV) is documented only in a single case in Ivory Coast [1,2].

From the large epidemics, we have learned that certain EVD outbreaks can be linked to individuals who recovered from the disease, even more than 5 years after infection [3,4]. Nevertheless, the majority of EVD outbreaks are most likely the result of independent spillover events from wildlife to humans. More than 45 years after the first descriptions of EVD outbreaks, the animal reservoir still remains elusive, but bats are considered as the most likely reservoir species. Multiple surveys of bats in West, Central and East Africa showed the presence of antibodies to orthoebolaviruses in at least eight frugivorous species and one insectivorous genus (*Mops* sp.) [5,6,7,8,9,10]. Despite large efforts from multiple groups, only a single study demonstrated the presence of viral RNA from Ebola virus (EBOV) in a handful of bats from three frugivorous bat species (*Epomops franqueti*, *Hypsignathus monstrosus* and *Myonycteris torquata*) during EVD outbreaks in Gabon and the Republic of Congo [11]. Ebola virus RNA has also been identified in apes in Gabon and the Republic of Congo, but the high mortality among ape populations indicate that they are not a reservoir but rather an intermediate animal host species [12,13,14]. Among other filoviruses, the role of bats as potential reservoir host species is well established. For example, a large diversity of orthomarburgviruses (MARV) have been amplified and sequenced from *Rousettus aegyptiacus* bats from different geographic regions [15,16,17,18,19]. Viral RNA from Bombali virus (BOMV) has been detected in insectivorous bats (*Mops condylurus* and *Chaerephon pumilus*) in East, Central and West Africa [20,21,22] and other filoviruses have been described in bats from Europe and Asia, like Lloviu virus and Reston virus (RESTV), respectively [23,24,25,26].

The low number of bats in which viral RNA was detected can be partially explained by the fact that orthoebolaviruses are most likely cleared from their hosts and can only be detected for a limited time period. Bats are infected with a wide diversity of potential pathogenic RNA viruses for humans [27] and seasonal variations in infection and immunological status have also been documented for other viral infections like henipaviruses, lyssaviruses, and coronaviruses in bats [5,28,29,30,31,32]. Temporal dynamics in viral shedding and seroprevalence have also been shown for Marburg virus [33]. We recently showed temporal variation of seroprevalence for orthoebolaviruses in a colony of *Eidolon helvum* bats in Yaoundé, the capital city of Cameroon [34]. Here, we extend and build further on our previous studies on antibodies to orthoebolaviruses in more than 9500 bats from Cameroon, DRC and Guinea, three countries with high potential for the emergence of orthoebolaviruses [35]. We explore the factors associated with the presence of antibodies to orthoebolaviruses.

## 2. Materials and Methods

### 2.1. Collection Sites

Between November 2015 and September 2020, samples were collected from free-ranging frugivorous and insectivorous bats in Cameroon, Guinea and the Democratic Republic of Congo (DRC). Bats were captured as previously described using mist nets of different mesh sizes or harp traps in roosting and foraging sites [9,34,36]. Nets or harp traps were set up before sunset and checked regularly for presence of bats. Venipuncture was performed on the propatagial or brachial vein and blood was subsequently dropped on Whatman 903 filter paper (GE Healthcare, Feasterville-Trevose, PA, USA) as previously described [9,34,36]. Samples were then air-dried and preserved individually as dried blood spots (DBS) in plastic bags containing silica desiccant and stored in hermetic boxes. Sample storage in the field was at ambient temperature for max 2–3 weeks and samples were then transferred to the central laboratory in each country to store them at −20 °C until analysis. After sampling, captured bats were released immediately. For each bat, morphological data were recorded (measurements of the body, the forearm, the tail, and the metacarpus of the third finger; weight; color) as well as sex, age class, and visual species identification. As described previously, the age of bats was classified according to their morphology and the development of their genitalia [34]. Late pregnancy and lactation in females were determined by palpation of the abdomen and the mammary glands and nipples [34]. The study was conducted in compliance with the national ethics committee from the DRC (ESP/CE/009/2020), Guinea (approval reference 074/CNERS/15, 26 November 2015) and Cameroon (N°2018/09/1090/CE/CNERSH/SP).

### 2.2. Screening for Orthoebolavirus Antibodies

Dried blood spots (DBS) were tested with a Luminex-based serological assay adapted for bats as previously described [9,34,36,37]. The assay included 10 commercially available recombinant orthoebolavirus proteins, glycoprotein (GP), nucleoprotein (NP) and viral protein 40 (VP40) from 4 different *Orthoebolavirus* species: EBOV, previously called Zaire (NP, amino acids [aa] 488 to 739, variant Mayinga 1976; VP40, aa 31 to 326, variant Kissidougou-Makona 2014; GP-k, aa 1 to 650, variant Kissidougou-Makona 2014; GP-m, aa 1 to 650, variant Mayinga 1976); SUDV, Sudan (NP, aa 361-738, variant Gulu; GP, aa 1 to 637, variant Uganda 2000; and VP40, aa 31 to 326, variant Gulu); BDBV, Bundibugyo (GP, aa 1 to 501, variant Uganda 2007; VP40, aa 31 to 326, variant Uganda 2007) and RESTV, Reston (GP, aa 1 to 650). Recombinant proteins were produced in insect cells and purchased from Sinobiologicals (Beijing, China) except for REST GP (IBT, Gaithersburg, MD). We first reconstituted blood from DBS as previously described [9,34,36,37] in 200 µL of incubation buffer. One hundred µL of sample, corresponding to the equivalent of a final plasma dilution of 1/2000, was incubated with 50 µL of magnetic beads coated with recombinant protein (2 μg protein/1.25 × 10^6^ beads) in 96-well flat-bottom chimney plates (Greiner bio one, Frickenhausen, Germany) on a plate shaker at 400 rpm for 16 h at 4 °C in the dark. After washing, 0.1 μg/mL of goat anti-bat biotin-labeled IgG (Euromedex, Souffelweyersheim, France) was added to each well and incubated for 30 min at 400 rpm at room temperature. After washing, we added 50 μL of 4 μg/mL streptavidin-R-phycoerythrin (Fisher Scientific/Life Technologies, Illkirch, France) per well and incubated for 10 min at 400 rpm at room temperature. Reactions were read with BioPlex-200 (BioRad, Marnes-la-Coquette, France) or MagPix (Luminex, Austin, TX, USA). At least 50 events were read for each bead set, and results were expressed as median fluorescence intensity (MFI) per 50 beads. Samples that showed positive signals were repeated in order to validate the results.

In the absence of well-characterized positive and/or negative control samples, the proportion of samples reactive for each antigen were calculated with previously defined stringent and non-stringent cut-off values [9,34,36]. First, we used the general formula based on the MFI values of negative control samples from European zoos, mean of the 145 negative samples plus 4 × standard deviation as described by De Nys and colleagues [9], as a non-stringent condition. Negative control samples were from captive-born insectivorous bat species in Europe (*Carollia perspicillata*, n = 103; *Pteropus giganteus*, n = 19; and *R. aegyptiacus*, n = 23) [9]. Secondly, we used the mean of cut-offs of three previously described statistical methods used in the absence of well-documented positive controls; i.e., change point analysis and fitted univariate binomial and exponential distributions at a 0.001 risk for error, as a stringent condition [34,36]. These cut-offs were described and calculated on a total of 8741 bats from Guinea, DRC and Cameroon, included in this study [36]. A sample was considered reactive with an antigen if the MFI value was above the cut-off. Analyses were performed with R version 4.3.0 software (https://www.r-project.org/, last accessed on 28 April 2023).

### 2.3. Molecular Confirmation of Bat Species

For a subset of samples, species identification recorded in the field was molecularly confirmed using the corresponding DBS sample as in our previous studies on viruses in bats [9,34,36]. At least one sample per sampling date, capture method and morphologic description at each site was confirmed. An 800 bp fragment of the mitochondrial cytochrome b (CytB) region was amplified using previously described primers, Cytb-L14724 (forward) and Cytb-H15506 (reverse) [38]. To increase PCR specificity for certain bat species, the forward primer was replaced by a newly designed forward primer Cytb-L1 5′-ATG ACC AAC ATC CGA AAA TCN CAC-3′ or Cytb-L2 5′-ATY TCY TCM TGA TGA AAY TTY GGM TC-3′ [36]. For samples that could not be readily amplified and/or sequenced in the CytB region, species identification was confirmed by amplifying a 386-bp mitochondrial DNA fragment of the 12S rRNA gene with primers 12S-L1091 and 12S-H1478 [39]. PCR products were directly sequenced with a BigDye Terminator version 3.1 sequencing kit (Life Technologies, Courtaboeuf, France) on an Applied Biosystems 3500 Genetic Analyzer (Thermo Fisher Scientific, Foster City, CA, USA). Sequences from both strands were reconstituted using the SeqMan Pro tool from the package DNAStar v17.0.2 (Lasergene, Madison, WI, USA). Sequences were uploaded in the NCBI BLAST web interface (https://blast.ncbi.nlm.nih.gov/Blast.cgi, accessed on 31 March 2023) to identify the most similar bat species. For sequences with no or low similarity (<97%) hits with species in Genbank, a phylogenetic tree was constructed using maximum likelihood methods implemented in PhyML with reference sequences in order to obtain genus identification [40]. Species identification was extrapolated for the remaining samples by combining molecular and field data. For certain insectivorous bats, especially from Molossidae, Rhinolophidae, Hipposideridae and Nycteridae families, identification was only possible at the genus level, mostly due to the lack of reference sequences in Genbank. For *Epomophorus gambiensis* and *Micropteropus pusillus*, species discrimination cannot be performed using only CytB sequences, and morphologic details on forearm and weight measurements were also used to discriminate the species, as previously described [36,41].

### 2.4. Statistical Analysis to Study Factors Associated with Seropositivity

All analyses were performed on Rstudio software version 2023.03.0+386. In order to have a synthetic overview of the data, calculations of raw seroprevalences and serological reactivity were carried out with the “binconf” function of the “Hmisc” package, specifying the “Wilson” method to obtain the seroprevalences and the 95% confidence intervals. The significance is obtained by a Fisher exact test using the “fisher.test” function, *p*-value < 0.05 was considered as significant. Secondly, we developed generalized linear mixed models (GLMM) with the “glmer” function from the “lme4” package. The aim is to test the influence of different factors on the probability of a bat presenting antibodies directed against GP Sudan. The reactivity to GP Sudan was analyzed as a binomial distribution. The explanatory variables are Species, Age, Country and Field session. These last two variables enabled the integration of a spatiotemporal effect in the model, to take into account the grouped samples collected on the same site and during the same sampling session. The final model chosen is the one with the lowest Akaike Information Criterion (AIC). Here, it is the model with the Species and Age variables as fixed effects and the Field session variable nested in the Country variable as a random effect. Odds ratios and 95% confidence intervals for each variable were obtained from the model.

## 3. Results

### 3.1. Bat Species and Sample Sites

**Figure 1:**
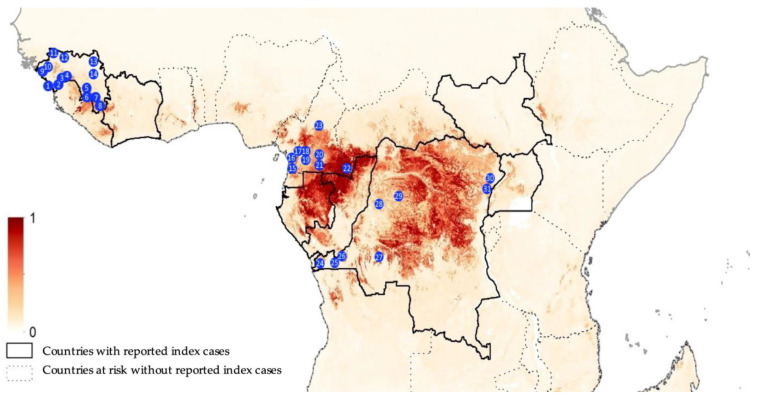
Collection sites from Guinea (1, Conakry; 2, Forecariah; 3, Kindia; 4, Mamou; 5, Kissidougou; 6, Guéckedou; 7, Macenta; 8, Nzérekoré; 9, Boffa; 10, Boke; 11, Koundara; 12, Mali; 13, Siguiri and 14, Kankan), Cameroon (15, Campo; 16, Bipindi; 17, Boumnyebel; 18, Yaoundé; 19, Mbalmayo; 20, Northern Periphery Dja; 21, Djoum; 22, Mambele and 23, Tibati) and Democratic Republic of Congo (24, Boma; 25, Kimpese; 26, Zongo; 27, Kikwit; 28, Bikoro; 29, Boende; 30, Komanda and 31, Beni). The map is adapted from Pigott et al. [35]; areas closer to dark red are estimated at highest risk for orthoebolavirus spillover events, and areas in light yellow are least at risk.

Overall, we analyzed blood samples from 9594 bats sampled between November 2015 and September 2020 in 31 different sites; 4886 samples in nine sites in Cameroon, 1764 samples in eight sites in the Democratic Republic of Congo and 2944 samples in 14 sites in Guinea (Figure 1). In total, 5972 frugivorous bats of at least eight different species and 3622 insectivorous bats of at least five different species were analyzed. For 3271 (34.1%) samples, species identification in the field was confirmed by sequence analysis. For some insectivorous bat families (Miniopteridae, Molossidae, Nycteridae, Rhinolophidae, Hipposideridae), identification was only possible at the genus level. For Molossidae bats, we could not always distinguish between *Mops* and *Chaerephon* genera, most likely because of the lack of sequences in GenBank, and we grouped both genera in the analysis (Table 1). Overall, 5767 samples have been reported in previous studies; i.e., surveys conducted between 2015 and 2017 between EVD outbreaks [9], surveys during EVD outbreaks in 2018 in DRC [36] and a one-year monitoring of an *Eidolon helvum* colony in Yaoundé, Cameroon [34]. The current study therefore adds new serological data on samples from 3827 bats, representing 39.9% of the total number of samples described in this study.

Among the 5972 frugivorous bats, *Eidolon helvum* (n = 1652; 27.7%), *Rousettus aegyptiacus* (n = 1140; 19.1%) and *Epomops* sp. (n = 1076; 18.02%) bats predominate, followed by *Epomophorus* sp. (n = 614; 10.3%), *Hypsignathus monstrosus* (n = 572; 9.6%), *Myonycteris torquata.* (n = 397; 6.6%), *Micropteropus pusillus* (n = 256; 4.3%), *Lissonycteris angolensis* (n = 186; 3.1%) and representatives of diverse other frugivorous species (n = 79; 1.3%; *Casinycteris* sp. (n = 18), *Megaloglossus woermanni* (n = 55), *Nanonycteris* sp. (n = 3) and *Scotonycteris* sp. (n = 3)) (Table 1). Two *Epomops* species were studied, i.e., *E*. *franqueti* in Cameroon and DRC (n = 1055), and *E*. *buetticoffi* (n = 21) in Guinea. Similarly, two *Epomophorus* species were included, related to their geographic range in Africa, i.e., *E*. *gambianus* in Guinea, Cameroon and western DRC (n = 348) and *E*. *labiatus* (n = 266) in eastern DRC. The 3622 insectivorous bats are largely dominated by *Hipposideros* species (43.9%) and bats from the Molossidae family (n = 1428; 39.4%; *Mops* and *Chaerephon* species) followed by *Miniopterus* sp. (n = 264; 7.3%), *Rhinolophus* sp. (n = 154; 4.3%), *Nycteris* sp. (2.4%) and representatives of diverse other insectivorous species (2.7%; *Coleura afra* (n = 6), *Glauconycteris* sp. (n = 8), *Kerivoula* sp. (n = 1), *Myotis* sp. (n = 4), *Neoromicia* sp. (n = 26), *Scotophilus* sp. (n = 45) and *Taphozous mauritianus* (n = 7) (Table 1).

Overall, 5080 (52.9%) bats were female, 4408 (45.9%) were male and for 106 (1.1%) sex was not recorded. The majority of bats were adults (n = 7873; 82.1%), 1186 (12.4%) were juveniles and for 535 (5.6%) information on age was not available. Only in Cameroon, the differentiation between adults and subadults was performed with 2959 (67.2%) adults, 438 (9.9%) subadults and 1007 (22.9%) juveniles for the 4404 bats for which age was recorded. Overall, late gestation or lactation was reported for 344 and 408 adult females, respectively.

### 3.2. Antibodies against Different Orthoebolavirus Antigens

**Figure 2:**
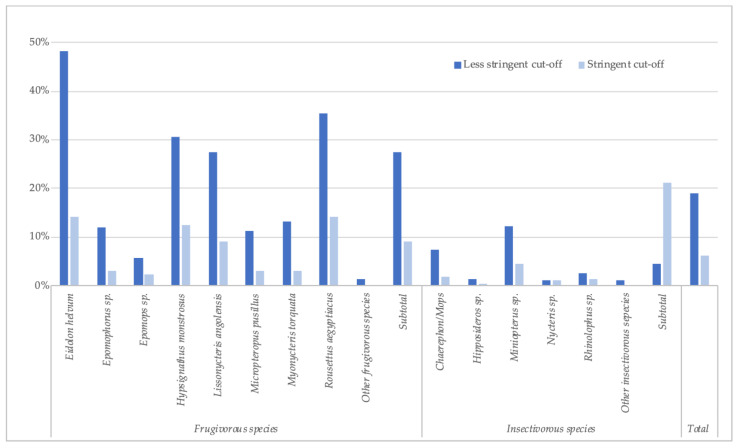
Percentage of samples reactive to at least one orthoebolavirus antigen (less stringent and stringent cut-off) for each bat species in Guinea, Cameroon and DRC.

Because of the absence of well-documented positive and negative samples from bats, we used stringent and less-stringent approaches to calculate the number and percentage of reactive samples, and, as in our previous reports, prevalence is thus expressed as a range (Table 2, Figure 2). The number of samples reacting with at least one antigen was 1810 (18.9%) by the less stringent cut-off (mean + 4SD) and 590 (6.1%) with the stringent cut-off. The highest reactivity was observed with GP antigens, especially for GP SUDV ranging between 2.0% and 14.0%, according to stringent or less-stringent cut-off, respectively (Table 2), followed by GP EBOV-k (2.1–10.6%), GP BDBV (1.5–9.1%), GP EBOV-m (1.9–5.8%) and only low reactivity and at lower MFIs was observed with GP RESTV (0.8–0.4%). The presence of antibodies to orthoebolavirus antigens varied per species and was higher in frugivorous than in insectivorous bats; 9.1–27.5% versus 1.3–4.6%, respectively. Antibodies to at least one antigen were observed in the eight predominant frugivorous species tested, with the highest reactivity seen in *Eidolon helvum* (14.1–48.1%) and *Rousettus aegyptiacus* (14.1–35.5%). In the three species in which EBOV RNA has been previously reported in Gabon and the Republic of Congo, antibodies were observed at variable levels, 12.4–30.6% for *Hypsignathus monstrosus*, 3.1–13.1% for *Myonycteris torquata*, and 2.2–5.8% for *Epomops* species. Antibodies were detected in *Epomops franqueti* and *E. buettikoferi* and in *Epomophorus gambianus* and *E*. *labiatus*. Among insectivorous species, antibody detection was highest in the *Chaerephon*/*Mops* and *Miniopterus* species, 1.8–7.5% and 4.5–12.1%, respectively.

Significantly less samples had antibodies to two or more antigens from the same *Orthoebolavirus* species (Table 3), i.e.; 0.2–1.4% for EBOV, 0.3–2.0% for SUDV and 0.0–0.1 for BDBV. The highest percentages were observed in *Eidolon helvum* and *Rousettus aegyptiacus*. In the other species, no or only sporadic cases were observed according to the stringency of cut-off values used. On the other hand, simultaneous reactivity to the same antigen from different *Orthoebolavirus* species was frequent, suggesting cross-reactivity between *Orthoebolavirus* species especially for GP proteins; 1.88–10.7% of blood samples were reactive to glycoprotein (GP) from more than two *Orthoebolavirus* species. Antibodies to VP-40 and NP of at least two *Orthoebolavirus* species were seen in 0.23–0.82% and 0.01–0.19% samples, respectively.

### 3.3. Factors Associated with Antibodies to Orthoebolaviruses

**Figure 3:**
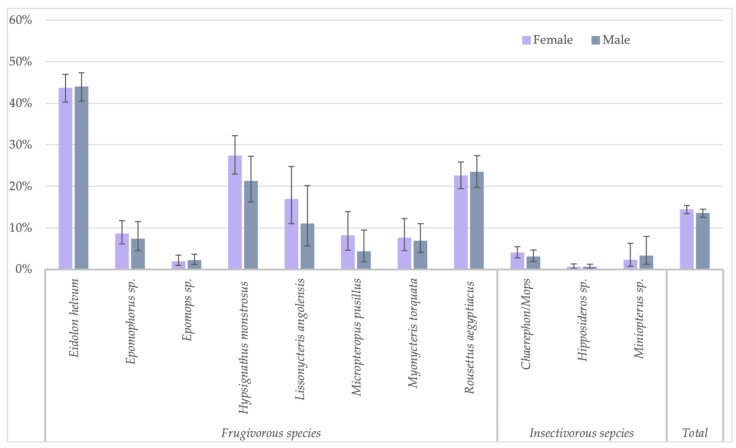
Percentage of samples reactive to GP SUDV (less stringent cut-off) and sex for the different bat species in Guinea, Cameroon and DRC.

**Figure 4:**
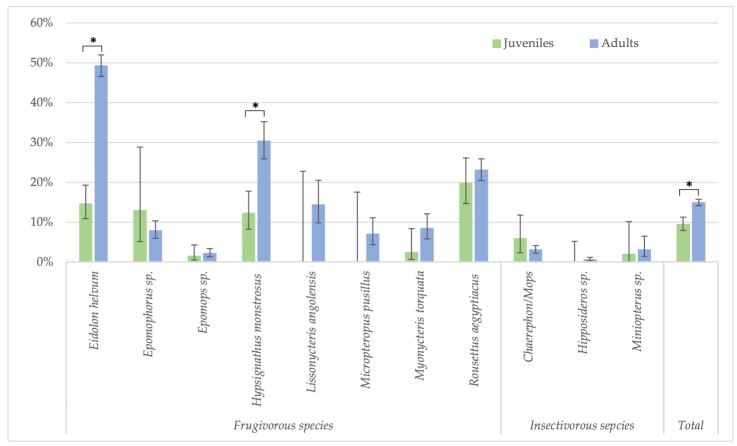
Percentage of samples reactive to GP SUDV (less stringent cut-off) and age (juveniles and adults) for the different bat species in Guinea, Cameroon and DRC. Symbols (*) represent significant differences (Fisher test) between juveniles/adults with *p*-value < 0.0001 for *Eidolon helvum*, *Hypsignathus monstrosus* and the total.

Because the highest reactivity was observed with GP SUDV, all further analyses on risk factors were performed for antibodies against this antigen for less stringent cut-offs. When significant differences were observed, they were also checked for stringent cut-off conditions. Seropositivity in females (730/5080; 14.4%) was slightly higher than in male (595/4408; 13.5%) bats (Fisher test: *p* < 0.22; Glmm: OR = 0.86 (CI 0.75–0.99), *p* < 0.03 for less-stringent cut-off and OR = 0.84 (CI 0.62–1.16, *p* = 0.3 for stringent cut-off) (Figure 3, Supplementary Table S1). Overall, juvenile bats were less likely to have antibodies as compared to adult bats, 9.4% (112/1186) versus 14.9% (1175/7873) (Fisher test: *p* < 0.0001; Glmm: OR = 0.36 (CI 0.28–0.45), *p* < 0.001) (Figure 4, Supplementary Table S2). The difference was observed for all bat species combined and remained significant at the individual species level for *Eidolon helvum* (Fisher test: *p* < 0.001) and *Hypsignathus monstrosus* (Fisher test: *p* < 0.0001) bats, for which high numbers of juvenile bats were collected. Only in *Epomophorus* species, an opposite trend was seen, but this can be related to the low number of juvenile samples. The same overall trend was observed with stringent cut-offs, but remained only significant for *Eidolon helvum* (Fisher test: *p* < 0.0005) but the trend was generally conserved at species level when sufficient samples were available for both age categories.

**Figure 5:**
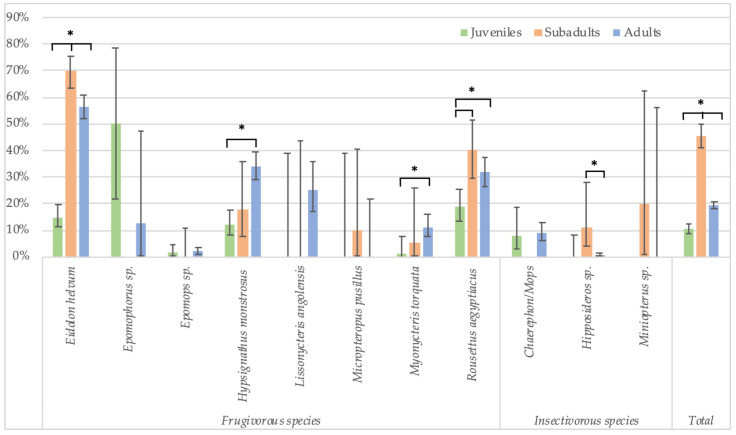
Percentage of samples reactive to GP SUDV (less stringent cut-off) and age (juveniles, subadults and adults) for the different bat species in Cameroon. Symbols (*) represent significant differences (Fisher test) between juveniles/subadults, juveniles/adults and subadults/adults for *Eidolon helvum (p* < 0.001); between juveniles/adults (*p* < 0.0001) for *Hypsignathus monstrosus*; between juveniles/adults (*p* = 0.01) for *Myonycteris torquata*; between juveniles/subadults (*p* < 0.001) and juveniles/adults (*p* = 0.002) for *Rousettus aegyptiacus*; between subadults/adults (*p* = 0.001) for *Hipposideros* sp.; and between juveniles/subadults, subadults/adults and juveniles/adults (*p* < 0.001) for the total species.

In Cameroon, adult bats were also classified as subadults and mature adults. These data show that seroprevalence is lowest in juveniles (10.5%, 106/1007), highest in subadults (45.4%, 199/438) and subsequently decreases in mature adults (19.2%, 569/2959) (Figure 5, Supplementary Table S3). The differences are significant for all species combined, and for both stringent (Fisher test: *p* < 0.0001 for juveniles versus subadults, and subadults versus adults; Glmm: respectively, OR = 0.23 (CI 0.16–0.33) and 0.6 (CI 0.45–0.8), *p* < 0.001) and non-stringent cut-off values (see legend of Figure 5 for Fisher test; Glmm: respectively, OR = 0.27 (CI 0.14–0.5) and 0.26 (CI 0.16–0.41), *p* < 0.001). At the species level, the differences are significant for *Eidolon helvum* and *Rousettus aegyptiacus* bats for which high numbers for each age category are available, and also for both stringent (Fisher test: *p* < 0.01 for all age categories of *E. helvum* and for juveniles and subadult *R. aegytiacus*) and non-stringent cut-offs (Fisher test: *p* < 0.01 for all age categories of *E. helvum* and for juvenile versus subadults and subadult versus adult *R. aegyptiacus*). For the other species, differences were not significant, most likely because sample numbers were too limited for certain age categories.

**Figure 6:**
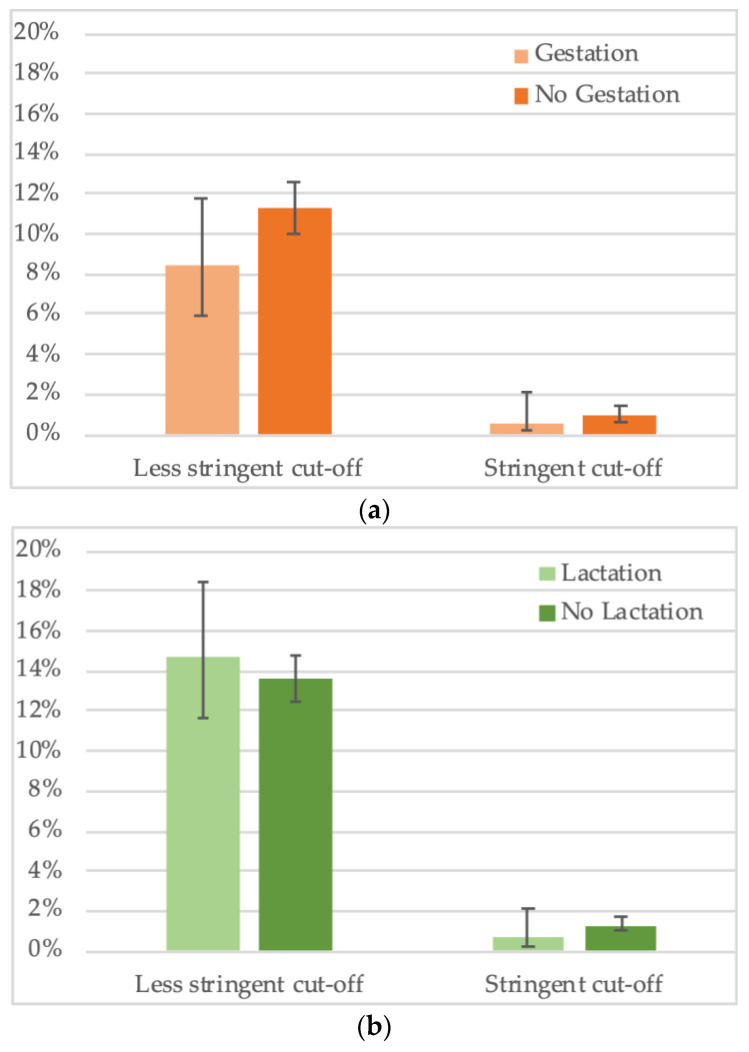
Percentage of samples reactive to GP SUDV (less stringent and stringent cut-off) and (**a**) gestation or (**b**) lactation status of adult female bats in Cameroon, Guinea and DRC.

For a total of 2794 female bats, gestation status was noted. We observed an overall trend for lower seropositivity in gestating females (8.4%, 29/344) versus non-gestating females (11.3%, 276/2450) (Figure 6a, Supplementary Table S4,) but the difference was not significant. Impact of gestation seems to vary according to species, for example, lower seroprevalence in gestating bats was observed for *Hypsignathus monstrosus* and *Lissonycteris angolensis*, the opposite was observed for *Eidolon helvum* and *Micropteropus pusillus* bats, and no difference was observed for *Rousettus aegyptiacus*. For the other species, insufficient sample numbers of gestating bats have been collected. For 3808 female bats with information on lactation status, no overall significant trend was seen, i.e., 14.7% (60/408) of lactating bats had antibodies versus 13.6% (462/3400) of non-lactating (Figure 6b, Supplementary Table S5). As for pregnancy, different trends were seen at the species level, for example, seroprevalence was lower in lactating bats for *Eidolon helvum*, *Hypsignathus monstrosus*, *Lissonycteris angolensis* and *Myonycteris torquata*. Overall, the data on reproductive stage need to be taken with caution, given the limited sample numbers per species.

## 4. Discussion

Comparison of orthoebolavirus seroprevalence in bats is difficult among different studies, because various antigens are used among assays and different methods are used to define cut-off values for seropositivity in the absence of well-characterized positive and negative control samples to validate assays. Here, we analyzed the presence of antibodies to different antigens from four *Orthoebolavirus* species in more than 9500 bats representing at least 8 frugivorous and 5 insectivorous bat species in Africa using the same methods. The samples have been collected over a 5-year period using the same techniques in the field, but more importantly also for antibody detection which thus allows comparisons per species and for factors that could be associated with presence of anti-orthoebolavirus antibodies. The bats were sampled in countries with known orthoebolavirus outbreaks (DRC and Guinea) or at risk for outbreaks (Cameroon) [35]. Compared to our previous studies, we analyzed more than 3800 new samples and increased significantly the numbers for insectivorous Molossidae bats (*Mops* and *Chaerephon* species) and for several frugivorous species like *Epomops franqueti*, *Hypsignathus monstrosus* and *Myonycteris torquata*, in which viral RNA of EBOV, responsible for the majority of EVD outbreaks in humans, has been identified [11]. We also tested more *Rousettus aegyptiacus* bats, known to be hosts for other filoviruses [16,18]. Overall, we confirm that antibodies are more frequently observed in frugivorous bats [9]. Among the eight frugivorous bat species with antibodies, the highest rates were seen in *Eidolon helvum*, *Rousettus aegyptiacus* and *Hypsignathus monstrosus* bats. We also observed antibodies, at lower but not negligible levels, in insectivorous bats from the Molossidae family (*Mops*/*Chaerephon*) and *Miniopterus* species. Among the three bat species in which EBOV RNA has been detected [11], only in *Hypsignathus monstrosus* high antibody levels were observed; *Myonycteris torquata* and *Epomops franqueti* species had lower seroprevalence rates.

We observed high proportions of bat samples that simultaneously reacted with GP antigens from more than one *Orthoebolavirus* species, suggesting cross-reactivity. Cross-reactivity was high among orthoebolaviruses that circulate in Africa and low reactivity was seen with the Reston Virus from Asia. Thus, this virus variant seems not to circulate in Africa. The same multiplex assay was used on plasma samples from humans who recovered from Ebola virus disease with EBOV in Guinea, and high rates of cross reactivity were observed among GP proteins from EBOV, SUDV and BDBV [42]. A study using convalescent sera from bats that have been experimentally infected with different *Orthoebolavirus* species also observed high cross-reactivity with another antibody assay [43]. Given the high cross-reactivity of antibodies to GP proteins, it can thus not be excluded that the observed antibodies correspond to cross-reactivity with other not-yet-identified orthoebolaviruses that circulate in bats. For example, the recent description of Bombali virus in Molosidae bats in Africa, as well as the presence of other filoviruses in bats from Asia, Europe and Africa, suggest that the genetic diversity of filoviruses in African bats can be high and is most likely underreported [16,17,18,20,21,22,23,24,25,26]. Moreover, not all filoviruses are pathogenic to humans, for example, Bombali and Reston orthoebolaviruses have not been documented in humans [44,45]. This could be the case for viruses that circulate in bat species in which high seroprevalence is seen, like *Eidolon helvum*. This bat species is widespread across Africa, where they live in large urban colonies and feed in fruit trees close to human habitats. Moreover, they are consumed as bushmeat in many regions, including in cities. Direct and indirect interactions between humans and *Eidolon helvum* bats are thus frequent and one could thus expect higher numbers of outbreaks, which is currently not the case. Nevertheless, false positive reactivity with other pathogens cannot be excluded. Many other studies, using different antibody assays and in other geographic areas, also showed the presence of antibodies to orthoebolaviruses in *Eidolon helvum* and in other bat species, even in regions where no outbreaks have been reported like Asia, Australia or the Caribbean Islands, suggesting that orthoebolaviruses or related viruses circulate in bats and could have a potential role in the ecology of orthoebolaviruses that are responsible for EVD outbreaks in humans [5,6,7,8,9,10,46,47,48,49,50].

We observed significant lower positivity rates in juvenile bats. Moreover, more in detailed analysis in Cameroon, where adult bats were also classified in subadult and mature adults, highest prevalence was observed in subadults and rates decreased subsequently in adults but remained higher than in juveniles. Thus, these observations extend our previous findings in an *Eidolon helvum* colony in Yaoundé to other bat species like *Rousettus aegyptiacus* and others [34]. Bats most likely become infected at the transition from the juvenile to the subadult stage. Viral shedding can thus be highest when this age category is highly present in bat colonies. Overall, this is also observed for other viruses in bats like Marburg, Hendraviruses, Coronaviruses, etc. [28,29,31,32,33,51]. Therefore, a close monitoring of bat ecology and population structure at a local scale is of paramount importance for understanding the dynamics of these viruses. We observed an overall, but not significant, trend for lower seroprevalence in pregnant bats and no difference for lactation. In contrast, Pourrut and colleagues observed higher prevalence in pregnant bats, but this could probably be explained by different species or sample numbers tested [6].

Our study, on more than 9500 bats, confirms clearly that age structure and reproductive phenology can play a role in filovirus infections in bats and corroborate the observations from mathematical models that reported a correlation between bat birthing and disease outbreaks [52,53]. Currently, no data are available on how orthoebolaviruses are transmitted within bat populations and how they are maintained in bat colonies. Moreover, data on experimentally infected bats are limited to the inoculation of only *Rousettus aegyptiacus* bats and these studies were not conclusive on the role of this bat species as a reservoir species for orthoebolaviruses, despite the presence of antibodies [54]. In humans, the main routes of viral transmission are direct contacts with infected body fluids from symptomatic or deceased patients, but viral relapse in EVD survivors can also be at the origin of new outbreaks [3,4]. The reasons for viral reactivation remain currently unknown, but could be related to decrease in antibody levels. Therefore, it can be possible that viral reactivation also occurs in bat populations and as such maintains viral circulation. Nevertheless, the immunological system of bats is different from humans and more studies are needed on viral circulation in bat populations.

Whereas it seems that frugivorous bats are more likely to be reservoirs of orthoebolaviruses, the role of insectivorous bats has also to be further examined, especially for Molossidae and *Miniopterus* bats. *Orthoebolavirus* fragments have potentially been detected in *Miniopterus* bats [55], although this needs to be confirmed. Mathematical models also suggest a role for Molosidae bats [53]. A recent study showed repeated establishment of persistent EBOV infections in primary cells from *M*. *condylurus* which might reflect the intrinsic ability that orthoebolaviruses may persist and be permanently maintained in this bat species [56]. In addition, analysis of *M. condylurus* genomic DNA samples revealed the presence of an Ebola virus nucleoprotein (NP)-derived pseudogene inserted in its genome and viral replication was observed in two species of Molosidae bats that were experimentally inoculated with EBOV [57,58].

Today, orthoebolaviruses are a significant public health problem because of the increasing number of outbreaks, the increasing number of infected individuals, the wide geographic spread of certain outbreaks and the high mortality rates despite availability of treatment and vaccine strategies. Overall, our data suggest that spill-over events are most likely to occur from young bats and that contact with these colonies should be avoided during the period of the year with highest presence of juveniles and young subadults bats to reduce the risk of spill-over events. Thus, not only more long-term studies are needed on colonies of bat species in which antibodies have been detected but these studies should also be performed in different geographic regions because for some species, birthing pulses vary according to latitudes and seasons [59].

Multidisciplinary studies on virological and ecological aspects combined with those on frequency and modes of contacts between humans and the different bat species, potentially harboring orthoebolaviruses, need to be conducted to consider all the different factors that play a role in spill-over events. From the above observations, it is also clear that when seroprevalence is compared in bats from different studies, differences can be related to different assays used but can also be due to population structure, which makes it even more challenging to compare data among different studies. Bats are increasingly hunted for consumption and exposure to infected organs is a more efficient transmission route than exposure to bat body fluids (saliva, urine, feces) [60,61,62,63]. It is also important to obtain more sequences from filoviruses in bats from Africa and to characterize them in more detail to evaluate their potential to infect human cells. These latter studies should most likely be completed at periods of the year when young bats are highly present in the bat colonies.

## Figures and Tables

**Table 1:** Number of different bat genera/species tested in each country (Guinea, Cameroon and DRC).

		Guinea	Cameroon	DRC	Total
**Frugivorous bats**					
**Pteropodidae**					
	*Eidolon helvum*	178	1020	454	1652
	*Epomophorus* sp. ^a^	406	19	189	614
	*Epomops* sp. ^b^	21	846	209	1076
	*Hypsignathus monstrosus*	18	542	12	572
	*Lissonycteris angolensis*	35	96	55	186
	*Micropteropus pusillus*	21	32	203	256
	*Myonycteris torquata*	1	294	102	397
	*Rousettus aegyptiacus*	551	584	5	1140
Other frugivorous species ^c^		3	44	32	79
**Subtotal**		**1234**	**3477**	**1261**	**5972**
**Insectivorous bats**					
**Molossidae**					
	*Chaerephon/Mops* ^d^	1039	368	21	1428
**Hipposideridae**					
	*Hipposideros* sp. ^d^	435	925	231	1591
**Miniopteridae**					
	*Miniopterus* sp. ^d^	46	9	209	264
**Nycteridae**					
	*Nycteris* sp. ^d^	79	7	2	88
**Rhinolophidae**					
	*Rhinolophus* sp. ^d^	74	71	9	154
Other insectivorous species ^e^		37	29	31	97
**Subtotal**		**1710**	**1409**	**503**	**3622**
**Total**		**2944**	**4886**	**1764**	**9594**

^a^ Two *Epomophorus* species were observed, *E*. *gambianus* in Guinea, Cameroon and western DRC and *E. labiatus* in eastern DRC. ^b^ Two *Epomops* species were observed, *E. franqueti* in Cameroon and DRC and *E*. *buettikoferi* in Guinea. ^c^ Other frugivorous species; *Casinycteris* sp. (n = 18), *Megaloglossus woermani* (n = 55), *Nanonycteris* sp. (n = 3) and *Scotonycteris* sp. (n = 3). ^d^ Identification at species level was not possible for a significant proportion of samples tested and were therefore grouped at the genus level. In the Molossidae family, differentiation between *Chaerephon* and *Mops* species was often not possible and they were analyzed together. ^e^ Other insectivorous species; *Coleura afra* (n = 6), *Glauconycteris* sp. (n = 8), *Kerivoula* sp. (n = 1), *Myotis* sp. (n = 4), *Neoromicia* sp. (n = 26), *Scotophilus* sp. (n = 45) and *Taphozous mauritianus* (n = 7).

**Table 2:** Number and percentage of samples reactive for each orthoebolavirus antigen used, expressed as a range from values obtained with stringent and less stringent cut-off values.

Genus/Species	N Tested	NP EBOV	GP EBOV-k	GP-EBOV-m	VP-EBOV	NP SUDV	GP SUDV	VP SUDV	GP BDBV	VP BDBV	GP RESTV	Total
		n (%)	n (%)	n (%)	n (%)	n (%)	n (%)	n (%)	n (%)	n (%)	n (%)	n (%)
**Frugivorous bats**												
**Pteropodidae**												
*Eidolon helvum*	1652	20–61 (1.2–3.7)	130–552 (7.9–33.4)	99–298 (6–18)	5–38 (0.3–2.3)	18–45 (1.1–2.7)	125–722 (7.6–43.7)	32–96 (1.9–5.8)	63–460 (3.8–27.8)	0–3 (0–0.2)	9–6 (0.5–0.4)	233–795 (14.1–48.1)
*Epomophorus* sp. ^a^	614	1–6 (0.2–1)	2–25 (0.3–4.1)	2–14 (0.3–2.3)	1–2 (0.2–0.3)	1–3 (0.2–0.5)	2–49 (0.3–8)	6–13 (1–2.1)	3–25 (0.5–4.1)	0–0 (0–0)	0–0 (0–0)	18–74 (2.9–12.1)
*Epomops* sp. ^b^	1076	5–7 (0.5–0.7)	1–7 (0.1–0.7)	2–4 (0.2–0.4)	4–14 (0.4–1.3)	7–8 (0.7–0.7)	0–21 (0–2)	6–12 (0.6–1.1)	1–8 (0.1–0.7)	1–3 (0.1–0.3)	1–0 (0.1–0)	24–62 (2.2–5.8)
*Hypsignathus monstrosus*	572	2–7 (0.3–1.2)	13–91 (2.3–15.9)	27–65 (4.7–11.4)	5–10 (0.9–1.7)	7–15 (1.2–2.6)	24–145 (4.2–25.3)	3–12 (0.5–2.1)	20–95 (3.5–16.6)	4–6 (0.7–1)	32–19 (5.6–3.3)	71–175 (12.4–30.6)
*Lissonycteris angolensis*	186	0–4 (0–2.2)	5–31 (2.7–16.7)	9–21 (4.8–11.3)	0–4 (0–2.2)	2–4 (1.1–2.2)	3–27 (1.6–14.5)	2–4 (1.1–2.2)	11–32 (5.9–17.2)	0–0 (0–0)	2–0 (1.1–0)	17–51 (9.1–27.4)
*Micropteropus pusillus*	256	2–2 (0.8–0.8)	2–13 (0.8–5.1)	1–7 (0.4–2.7)	0–0 (0–0)	2–4 (0.8–1.6)	1–16 (0.4–6.3)	2–5 (0.8–2)	1–7 (0.4–2.7)	0–0 (0–0)	0–0 (0–0)	8–29 (3.1–11.3)
*Myonycteris torquata*	397	0–1 (0–0.3)	4–20 (1–5)	2–12 (0.5–3)	0–5 (0–1.3)	3–7 (0.8–1.8)	1–29 (0.3–7.3)	3–7 (0.8–1.8)	1–16 (0.3–4)	1–1 (0.3–0.3)	0–0 (0–0)	12–52 (3–13.1)
*Rousettus aegyptiacus*	1140	14–54 (1.2–4.7)	36–189 (3.2–16.6)	36–93 (3.2–8.2)	32–90 (2.8–7.9)	43–71 (3.8–6.2)	32–261 (2.8–22.9)	41–92 (3.6–8.1)	31–162 (2.7–14.2)	9–18 (0.8–1.6)	20–10 (1.8–0.9)	160–405 (14–35.5)
Other frugivorous species ^c^	79	0–0 (0–0)	0–0 (0–0)	0–0 (0–0)	0–0 (0–0)	0–1 (0–1.3)	0–0 (0–0)	0–0 (0–0)	0–0 (0–0)	0–0 (0–0)	0–0 (0–0)	0–1 (0–1.3)
**Subtotal**	**5972**	**44–142 (0.7–2.4)**	**193–928 (3.2–15.5)**	**178–514 (3–8.6)**	**47–163 (0.8–2.7)**	**83–158 (1.4–2.6)**	**188–1270 (3.1–21.3)**	**95–241 (1.6–4)**	**131–805 (2.2–13.5)**	**15–31 (0.3–0.5)**	**64–35 (1.1–0.6)**	**543–1644 (9.1–27.5)**
**Insectivorous bats**												
**Molossidae**												
*Chaerephon/Mops* ^d^	1428	4–15 (0.3–1.1)	9–70 (0.6–4.9)	7–32 (0.5–2.2)	3–9 (0.2–0.6)	1–6 (0.1–0.4)	1–54 (0.1–3.8)	5–12 (0.4–0.8)	7–51 (0.5–3.6)	1–1 (0.1–0.1)	10–2 (0.7–0.1)	26–107 (1.8–7.5)
**Hipposideridae**												
*Hipposideros* sp. ^d^	1591	0–0 (0–0)	1–7 (0.1–0.4)	0–3 (0–0.2)	1–2 (0.1–0.1)	3–7 (0.2–0.4)	0–8 (0–0.5)	2–3 (0.1–0.2)	0–7 (0–0.4)	0–0 (0–0)	1–0 (0.1–0)	6–21 (0.4–1.3)
**Miniopteridae**												
*Miniopterus* sp. ^d^	264	1–1 (0.4–0.4)	1–11 (0.4–4.2)	1–5 (0.4–1.9)	6–11 (2.3–4.2)	1–4 (0.4–1.5)	0–7 (0–2.7)	3–7 (1.1–2.7)	1–9 (0.4–3.4)	0–1 (0–0.4)	3–1 (1.1–0.4)	12–32 (4.5–12.1)
**Nycteridae**												
*Nycteris* sp. ^d^	88	0–0 (0–0)	0–0 (0–0)	0–1 (0–1.1)	0–0 (0–0)	0–0 (0–0)	0–0 (0–0)	0–0 (0–0)	0–0 (0–0)	0–0 (0–0)	0–0 (0–0)	1–1 (1.1–1.1)
**Rhinolophidae**												
*Rhinolophus* sp. ^d^	154	0–1 (0–0.6)	0–0 (0–0)	0–0 (0–0)	1–1 (0.6–0.6)	0–0 (0–0)	0–0 (0–0)	2–3 (1.3–1.9)	0–0 (0–0)	0–0 (0–0)	0–0 (0–0)	2–4 (1.3–2.6)
Other insectivorous species ^e^	97	0–0 (0–0)	0–0 (0–0)	0–0 (0–0)	0–1 (0–1)	0–0 (0–0)	0–0 (0–0)	0–0 (0–0)	0–0 (0–0)	0–0 (0–0)	0–0 (0–0)	0–1 (0–1)
**Subtotal**	**3622**	**5–17 (0.1–0.5)**	**11–88 (0.3–2.4)**	**8–41 (0.2–1.1)**	**11–24 (0.3–0.7)**	**5–17 (0.1–0.5)**	**1–69 (0–1.9)**	**12–25 (0.3–0.7)**	**8–67 (0.2–1.8)**	**1–2 (0–0.1)**	**14–3 (0.4–0.1)**	**47–166 (1.3–4.6)**
**Total**	**9594**	**49–159** **(0.5–1.7)**	**204–1016** **(2.1–10.6)**	**186–555** **(1.9–5.8)**	**58–187** **(0.6–1.9)**	**88–175** **(0.9–1.8)**	**189–1339** **(2–14)**	**107–266** **(1.1–2.8)**	**139–872** **(1.4–9.1)**	**16–33** **(0.2–0.3)**	**78–38** **(0.8–0.4)**	**590–1810** **(6.1–18.9)**

^a^ Two *Epomophorus* species were observed, *E*. *gambianus* in Guinea, Cameroon and western DRC and *E. labiatus* in eastern DRC. ^b^ Two *Epomops* species were observed, *E. franqueti* in Cameroon and DRC and *E*. *buettikoferi* in Guinea. ^c^ Other frugivorous species; *Casinycteris* sp. (n = 18), *Megaloglossus woermani* (n = 55), *Nanonycteris* sp. (n = 3) and *Scotonycteris* sp. (n = 3). ^d^ Identification at species level was not possible for a significant proportion of samples tested and were therefore grouped at the genus level. In the Molossidae family, differentiation between *Chaerephon* and *Mops* species was often not possible and they were analyzed together. ^e^ Other insectivorous species; *Coleura afra* (n = 6), *Glauconycteris* sp.(n = 8), *Kerivoula* sp. (n = 1), *Myotis* sp. (n = 4), *Neoromicia* sp. (n = 26), *Scotophilus* sp. (n = 45) and *Taphozous mauritianus* (n = 7).

**Table 3:** Number and percentage of samples reactive to at least two orthoebolavirus antigens from the same *Orthoebolavirus* species, expressed as a range from values obtained with stringent and less stringent cut-off values.

Genus/Species	N Tested	NP + GP EBOV	NP + VP EBOV	GP + VP EBOV	NP + GP + VP EBOV	Total EBOV *	NP + GP SUDV	NP + VP SUDV	GP + VP SUDV	NP + GP + VP SUDV	Total SUDV*	GP + VP BDBV	Total **
		n (%)	n (%)	n (%)	n (%)	n (%)	n (%)	n (%)	n (%)	n (%)	n (%)	n (%)	n (%)
**Frugivorous bats**													
**Pteropodidae**													
*Eidolon helvum*	1652	3–28 (0.2–1.7)	1–2 (0.1–0.1)	0–18 (0–1.1)	0–6 (0–0.4)	4–54 (0.2–3.3)	1–22 (0.1–1.3)	0–0 (0–0)	9–72 (0.5–4.4)	0–3 (0–0.2)	10–97 (0.6–5.9)	0–2 (0–0.1)	13–128 (0.8–7.7)
*Epomophorus* sp. ^a^	614	0–1 (0–0.2)	0–0 (0–0)	0–0 (0–0)	0–0 (0–0)	0–1 (0–0.2)	0–2 (0–0.3)	0–0 (0–0)	0–2 (0–0.3)	0–0 (0–0.2)	0–4 (0–0.7)	0–0 (0–0.1)	0–5 (0–0.8)
*Epomops* sp. ^b^	1076	0–0 (0–0)	0–0 (0–0)	0–1 (0–0.1)	0–0 (0–0)	0–1 (0–0.1)	0–0 (0–0)	2–2 (0.2–0.2)	0–0 (0–0)	0–0 (0–0.2)	2–2 (0.2–0.2)	0–0 (0–0.1)	2–3 (0.2–0.3)
*Hypsignathus monstrosus*	572	1–4 (0.2–0.7)	0–0 (0–0)	0–2 (0–0.3)	0–0 (0–0)	1–6 (0.2–1)	0–8 (0–1.4)	0–0 (0–0)	0–6 (0–1)	0–0 (0–0.2)	0–14 (0–2.4)	0–0 (0–0.1)	1–20 (0.2–3.5)
*Lissonycteris angolensis*	186	0–2 (0–1.1)	0–0 (0–0)	0–1 (0–0.5)	0–0 (0–0)	0–3 (0–1.6)	0–0 (0–0)	0–0 (0–0)	0–1 (0–0.5)	0–0 (0–0)	0–1 (0–0.5)	0–0 (0–0.1)	0–4 (0–2.2)
*Micropteropus pusillus*	256	0–0 (0–0)	0–0 (0–0)	0– (0–0)	0–0 (0–0)	0–0 (0–0)	0–1 (0–0.4)	0–0 (0–0)	0–0 (0–0)	0–0 (0–0.2)	0–1 (0–0.4)	0–0 (0–0.1)	0–1 (0–0.4)
*Myonycteris torquata*	397	0–0 (0–0)	0–0 (0–0)	0–1 (0–0.3)	0–0 (0–0)	0–1 (0–0.3)	0–0 (0–0)	0–0 (0–0)	0–0 (0–0)	0–0 (0–0.2)	0–0 (0–0)	0–0 (0–0.1)	0–1 (0–0.3)
*Rousettus aegyptiacus*	1140	1–13 (0.1–1.1)	2–5 (0.2–0.4)	6–30 (0.5–2.6)	0–14 (0–1.2)	9–62 (0.8–5.4)	2–16 (0.2–1.4)	5–11 (0.4–1)	6–28 (0.5–2.5)	1–14 (0.1–1.2)	14–69 (1.2–6.1)	0–9 (0–0.8)	20–103 (1.8–9)
Other frugivorous species ^c^	79	0–0 (0–0)	0–0 (0–0)	0–0 (0–0)	0–0 (0–0)	0–0 (0–0)	0–0 (0–0)	0–0 (0–0)	0–0 (0–0)	0–0 (0–0)	0–0 (0–0)	0–0 (0–0)	0–0 (0–0)
**Subtotal**	**5972**	**5–48 (0.1–0.8)**	**3–7 (0.1–0.1)**	**6–53 (0.1–0.9)**	**0–20 (0–0.3)**	**14–128 (0.2–2.1)**	**3–49 (0.1–0.8)**	**7–13 (0.1–0.2)**	**15–109 (0.3–1.8)**	**1–17 (0–0.3)**	**26–188 (0.4–3.1)**	**0–11 (0–0.2)**	**36–265 (0.6–4.4)**
**Insectivorous bats**													
**Molossidae**													
*Chaerephon/Mops* ^d^	1428	1–4 (0.1–0.3)	2–1 (0.1–0.1)	1–2 (0.1–0.1)	0–1 (0–0.1)	4–8 (0.3–0.6)	0–2 (0–0.1)	0–0 (0–0)	0–3 (0–0.2)	0–0 (0–0)	0–5 (0–0.4)	0–0 (0–0)	4–12 (0.3–0.8)
**Hipposideridae**													
*Hipposideros* sp. ^d^	1591	0–0 (0–0)	0–0 (0–0)	1–1 (0.1–0.1)	0–0 (0–0)	1–1 (0.1–0.1)	0–0 (0–0)	0–0 (0–0)	0–0 (0–0)	0–1 (0–0.1)	0–1 (0–0.1)	0–0 (0–0)	1–1 (0.1–0.1)
**Miniopteridae**													
*Miniopterus* sp. ^d^	264	0–0 (0–0)	1–1 (0.4–0.4)	0–1 (0–0.4)	0–0 (0–0)	1–2 (0.4–0.8)	0–0 (0–0)	0–0 (0–0)	0–0 (0–0)	0–0 (0–0)	0–0 (0–0)	0–0 (0–0)	1–3 (0.4–1.1)
**Nycteridae**													
*Nycteris* sp. ^d^	88	0–0 (0–0)	0–0 (0–0)	0–0 (0–0)	0–0 (0–0)	0–0 (0–0)	0–0 (0–0)	0–0 (0–0)	0–0 (0–0)	0–0 (0–0)	0–0 (0–0)	0–0 (0–0)	0–0 (0–0)
**Rhinolophidae**													
*Rhinolophus* sp. ^d^	154	0–0 (0–0)	0–0 (0–0)	0–0 (0–0)	0–0 (0–0)	0–0 (0–0)	0–0 (0–0)	0–0 (0–0)	0–0 (0–0)	0–0 (0–0)	0–0 (0–0)	0–0 (0–0)	0–0 (0–0)
Other insectivorous species ^e^	97	0–0 (0–0)	0–0 (0–0)	0–0 (0–0)	0–0 (0–0)	0–0 (0–0)	0–0 (0–0)	0–0 (0–0)	0–0 (0–0)	0–0 (0–0)	0–0 (0–0)	0–0 (0–0)	0–0 (0–0)
**Subtotal**	**3622**	**1–4 (0–0.1)**	**3–2 (0.1–0.1)**	**2–4 (0.1–0.1)**	**0–1 (0–0)**	**6–11 (0.2–0.3)**	**0–2 (0–0.1)**	**0–0 (0–0)**	**0–3 (0–0.1)**	**0–1 (0–0)**	**0–6 (0–0.2)**	**0–0 (0–0)**	**6–16 (0.2–0.4)**
**Total**	**9594**	**6–52 (0.1–0.5)**	**6–9 (0.1–0.1)**	**8–57 (0.1–0.6)**	**0–21 (0–0.2)**	**20–139 (0.2–1.4)**	**3–51 (0–0.5)**	**7–13 (0.1–0.1)**	**15–112 (0.2–1.2)**	**1–18 (0–0.2)**	**26–194 (0.3–2)**	**0–11 (0–0.1)**	**46–281 (0.5–2.9)**

* corresponds to the sum of the number of samples positive for at least 2 different antigens from the same *Orthoebolavirus* species. ** corresponds to the number of samples positive for at least 2 different antigens from the same lineage and also for 2 different antigens from another lineage (for example if a sample is positive for NP + GP EBOV and VP+ GP SUDV then it is only counted once in the total). ^a^ Two *Epomophorus* species were observed, *E*. *gambianus* in Guinea, Cameroon and western DRC and *E. labiatus* in eastern DRC. ^b^ Two *Epomops* species were observed, *E. franqueti* in Cameroon and DRC and *E*. *buettikoferi* in Guinea. ^c^ Other frugivorous species; *Casinycteris* sp. (n = 18), *Megaloglossus woermani* (n = 55), *Nanonycteris* sp. (n = 3) and *Scotonycteris* sp. (n = 3). ^d^ Identification at species level was not possible for a significant proportion of samples tested and were therefore grouped at the genus level. In the Molossidae family, differentiation between *Chaerephon* and *Mops* species was often not possible and they were analyzed together. ^e^ Other insectivorous species; *Coleura afra* (n = 6), *Glauconycteris* sp. (n = 8), *Kerivoula* sp. (n = 1), *Myotis* sp. (n = 4), *Neoromicia* sp. (n = 26), *Scotophilus* sp. (n = 45) and *Taphozous mauritianus* (n = 7).

## Data Availability

Data are available upon request, and data on bat sampling are available on the EBO-SURSY website (https://rr-africa.woah.org/en/projects/ebo-sursy-en/, accessed on 13 July 2023).

## Supplementary Materials

The following supporting information can be downloaded at: https://www.mdpi.com/article/10.3390/v15091927/s1, Table S1: seroprevalence of IgG antibodies to GP SUDV (less stringent cut-off) according to sex for the different bat species in Guinea, Cameroon and DRC. Table S2: seroprevalence of IgG antibodies to GP SUDV (less stringent cut-off) for juveniles and adults (subadults and mature adults) for the different bat species in Guinea, Cameroon and DRC. Table S3: seroprevalence of IgG antibodies to GP SUDV (less stringent cut-off) for juveniles, subadults and adults for the different bat species in Cameroon. Table S4: seroprevalence of IgG antibodies to GP SUDV (less stringent cut-off) for gestating and non-gestating adult female bats for the different bat species in Guinea, Cameroon and DRC; Table S5: seroprevalence of IgG antibodies to GP SUDV (less stringent cut-off) for lactating and non-lactating adult female bats for the different bat species in Guinea, Cameroon and DRC.

## References

[1] CDC History of Ebola Virus Disease (EVD) Outbreaks. https://www.cdc.gov/vhf/ebola/history/chronology.html.

[2] Biedenkopf N., Bukreyev A., Chandran K., Di Paola N., Formenty P.B.H., Griffiths A., Hume A.J., Mühlberger E., Netesov S.V., Palacios G. (2023). Renaming of genera *Ebolavirus* and *Marburgvirus* to *Orthoebolavirus* and *Orthomarburgvirus*, respectively, and introduction of binomial species names within family Filoviridae. Arch. Virol..

[3] Keita A.K., Koundouno F.R., Faye M., Düx A., Hinzmann J., Diallo H., Ayouba A., Le Marcis F., Soropogui B., Ifono K. (2021). Resurgence of Ebola virus in 2021 in Guinea suggests a new paradigm for outbreaks. Nature.

[4] Mbala-Kingebeni P., Pratt C., Mutafali-Ruffin M., Pauthner M.G., Bile F., Nkuba-Ndaye A., Black A., Kinganda-Lusamaki E., Faye M., Aziza A. (2021). Ebola Virus Transmission Initiated by Relapse of Systemic Ebola Virus Disease. N. Engl. J. Med..

[5] Pourrut X., Délicat A., Rollin P.E., Ksiazek T.G., Gonzalez J.-P., Leroy E.M. (2007). Spatial and Temporal Patterns of Zaire Ebolavirus Antibody Prevalence in the Possible Reservoir Bat Species. J. Infect. Dis..

[6] Pourrut X., Souris M., Towner J.S., Rollin P.E., Nichol S.T., Gonzalez J.-P., Leroy E. (2009). Large Serological Survey Showing Cocirculation of Ebola and Marburg Viruses in Gabonese Bat Populations; and a High Seroprevalence of Both Viruses in *Rousettus aegyptiacus*. BMC Infect. Dis..

[7] Hayman D.T.S., Yu M., Crameri G., Wang L.-F., Suu-Ire R., Wood J.L.N., Cunningham A.A. (2012). Ebola Virus Antibodies in Fruit Bats; Ghana; West Africa. Emerg. Infect. Dis..

[8] Ogawa H., Miyamoto H., Nakayama E., Yoshida R., Nakamura I., Sawa H., Ishii A., Thomas Y., Nakagawa E., Matsuno K. (2015). Seroepidemiological Prevalence of Multiple Species of Filoviruses in Fruit Bats (*Eidolon helvum*) Migrating in Africa. J. Infect. Dis..

[9] De Nys H.M., Kingebeni P.M., Keita A.K., Butel C., Thaurignac G., Villabona-Arenas C.J., Lemarcis T., Geraerts M., Vidal N., Esteban A. (2018). Survey of Ebola Viruses in Frugivorous and Insectivorous Bats in Guinea; Cameroon; and the Democratic Republic of the Congo; 2015–2017. Emerg. Infect. Dis..

[10] Seifert S.N., Fischer R.J., Kuisma E., Badzi Nkoua C., Bounga G., Akongo M.J., Schulz J.E., Escudero-Pérez B., Akoundzie B.J., Ampiri V.R.B. (2022). Zaire ebolavirus surveillance near the Bikoro region of the Democratic Republic of the Congo during the 2018 outbreak reveals presence of seropositive bats. PLoS Negl. Trop. Dis..

[11] Leroy E.M., Kumulungui B., Pourrut X., Rouquet P., Hassanin A., Yaba P., Délicat A., Paweska J.T., Gonzalez J.-P., Swanepoel R. (2005). Fruit bats as reservoirs of Ebola virus. Nature.

[12] Leroy E.M., Rouquet P., Formenty P., Souquière S., Kilbourne A., Froment J.M., Bermejo M., Smit S., Karesh W., Swanepoel R. (2004). Multiple Ebola virus transmission events and rapid decline of central African wildlife. Science.

[13] Rouquet P., Froment J.M., Bermejo M., Kilbourn A., Karesh W., Reed P., Kumulungui B., Yaba P., Délicat A., Rollin P.E. (2005). Wild animal mortality monitoring and human Ebola outbreaks; Gabon and Republic of Congo; 2001–2003. Emerg. Infect. Dis..

[14] Bermejo M., Rodríguez-Teijeiro J.D., Illera G., Barroso A., Vilà C., Walsh P.D. (2006). Ebola outbreak killed 5000 gorillas. Science.

[15] Towner J.S., Pourrut X., Albariño C.G., Nkogue C.N., Bird B.H., Grard G., Ksiazek T.G., Gonzalez J.-P., Nichol S.T., Leroy E.M. (2007). Marburg Virus Infection Detected in a Common African Bat. PLoS ONE.

[16] Towner J.S., Amman B.R., Sealy T.K., Carroll S.A.R., Comer J.A., Kemp A., Swanepoel R., Paddock C.D., Balinandi S., Khristova M.L. (2009). Isolation of Genetically Diverse Marburg Viruses from Egyptian Fruit Bats. PLoS Pathog..

[17] Amman B.R., Nyakarahuka L., McElroy A.K., Dodd K.A., Sealy T.K., Schuh A.J., Shoemaker T.R., Balinandi S., Atimnedi P., Kaboyo W. (2014). Marburgvirus Resurgence in Kitaka Mine Bat Population after Extermination Attempts; Uganda. Emerg. Infect. Dis..

[18] Amman B.R., Bird B.H., Bakarr I.A., Bangura J., Schuh A.J., Johnny J., Sealy T.K., Conteh I., Koroma A.H., Foday I. (2020). Isolation of Angola-like Marburg Virus from Egyptian Rousette Bats from West Africa. Nat. Commun..

[19] Makenov M.T., Boumbaly S., Tolno F.R., Sacko N., N’Fatoma L.T., Mansare O., Kolie B., Stukolova O.A., Morozkin E.S., Kholodilov I.S. (2023). Marburg virus in Egyptian Rousettus bats in Guinea: Investigation of Marburg virus outbreak origin in 2021. PLoS Negl. Trop. Dis..

[20] Goldstein T., Anthony S.J., Gbakima A., Bird B.H., Bangura J., Tremeau-Bravard A., Belaganahalli M.N., Wells H.L., Dhanota J.K., Liang E. (2018). The Discovery of Bombali Virus Adds Further Support for Bats as Hosts of Ebolaviruses. Nat. Microbiol..

[21] Forbes K.M., Al K.M.F.E., Jääskeläinen A.J., Abdurahman S., Ogola J., Masika M.M., Kivistö I., Alburkat H., Plyusnin I., Levanov L. (2019). Bombali Ebola Virus in *Mops condylurus* Bat; Kenya. Emerg. Infect. Dis..

[22] Karan L.S., Makenov M.T., Korneev M.G., Sacko N., Boumbaly S., Yakovlev S.A., Kourouma K., Bayandin R.B., Gladysheva A.V., Shipovalov A.V. (2019). Bombali Virus in *Mops condylurus* Bats; Guinea. Emerg. Infect. Dis..

[23] Negredo A., Palacios G., Vázquez-Morón S., González F., Dopazo H., Molero F., Juste J., Quetglas J., Savji N., Martínez M.D.L.C. (2011). Discovery of an Ebolavirus-Like Filovirus in Europe. PLoS Pathog..

[24] Jayme S.I., Field H.E., de Jong C., Olival K.J., Marsh G., Tagtag A.M., Hughes T., Bucad A.C., Barr J., Azul R.R. (2015). Molecular evidence of Ebola Reston virus infection in Philippine bats. Virol. J..

[25] Yang X.-L., Zhang Y.-Z., Jiang R.-D., Guo H., Zhang W., Li B., Wang N., Wang L., Waruhiu C., Zhou J.-H. (2017). Genetically Diverse Filoviruses in *Rousettus* and *Eonycteris* spp. Bats; China; 2009 and 2015. Emerg. Infect. Dis..

[26] Kemenesi G., Kurucz K., Dallos B., Zana B., Földes F., Boldogh S., Görföl T., Carroll M.W., Jakab F. (2018). Re-emergence of Lloviu virus in *Miniopterus schreibersii* bats; Hungary; 2016. Emerg. Microbes Infect..

[27] Hayman D.T. (2016). Bats as Viral Reservoirs. Annu. Rev. Virol..

[28] Gloza-Rausch F., Ipsen A., Seebens A., Göttsche M., Panning M., Drexler J.F., Petersen N., Annan A., Grywna K., Müller M. (2008). Detection and Prevalence Patterns of Group I Coronaviruses in Bats; Northern Germany. Emerg. Infect. Dis..

[29] Plowright R.K., Field H.E., Smith C., Divljan A., Palmer C., Tabor G., Daszak P., Foley J.E. (2008). Reproduction and nutritional stress are risk factors for Hendra virus infection in little red flying foxes (*Pteropus scapulatus*). Proc. R. Soc. B Biol. Sci..

[30] Dietrich M., Wilkinson D.A., Benlali A., Lagadec E., Ramasindrazana B., Dellagi K., Tortosa P. (2015). Leptospira and paramyxovirus infection dynamics in a bat maternity enlightens pathogen maintenance in wildlife. Environ. Microbiol..

[31] Montecino-Latorre D., Goldstein T., Gilardi K., Wolking D., Van Wormer E., Kazwala R., Ssebide B., Nziza J., Sijali Z., Cranfield M. (2020). Reproduction of East-African bats may guide risk mitigation for coronavirus spillover. One Health Outlook.

[32] Meta Djomsi D., Lacroix A., Soumah A.K., Kinganda Lusamaki E., Mesdour A., Raulino R., Esteban A., Ndong Bass I., Mba Djonzo F.A., Goumou S. (2023). Coronaviruses Are Abundant and Genetically Diverse in West and Central African Bats; including Viruses Closely Related to Human Coronaviruses. Viruses.

[33] Amman B.R., Carroll S.A., Reed Z.D., Sealy T.K., Balinandi S., Swanepoel R., Kemp A., Erickson B.R., Comer J.A., Campbell S. (2012). Seasonal Pulses of Marburg Virus Circulation in Juvenile *Rousettus aegyptiacus* Bats Coincide with Periods of Increased Risk of Human Infection. PLoS Pathog..

[34] Djomsi D.M., Mba Djonzo F.A., Ndong Bass I., Champagne M., Lacroix A., Thaurignac G., Esteban A., De Nys H., Bourgarel M., Akoachere J.F. (2022). Dynamics of Antibodies to Ebolaviruses in an *Eidolon helvum* Bat Colony in Cameroon. Viruses.

[35] Pigott D.M., Millear A.I., Earl L., Morozoff C., Han B.A., Shearer F.M., Weiss D.J., Brady O.J., Kraemer M.U., Moyes C.L. (2016). Updates to the zoonotic niche map of Ebola virus disease in Africa. Elife.

[36] Lacroix A., Mbala Kingebeni P., Ndimbo Kumugo S.P., Lempu G., Butel C., Serrano L., Vidal N., Thaurignac G., Esteban A., Mukadi Bamuleka D. (2021). Investigating the Circulation of Ebola Viruses in Bats during the Ebola Virus Disease Outbreaks in the Equateur and North Kivu Provinces of the Democratic Republic of Congo from 2018. Pathogens.

[37] Ayouba A., Touré A., Butel C., Keita A.K., Binetruy F., Sow M.S., Foulongne V., Delaporte E., Peeters M. (2017). Development of a Sensitive and Specific Serological Assay Based on Luminex Technology for Detection of Antibodies to Zaire Ebola Virus. J. Clin. Microbiol..

[38] Irwin D.M., Kocher T.D., Wilson A.C. (1991). Evolution of the Cytochrome b Gene of Mammals. J. Mol. Evol..

[39] Van der Kuyl A.C., Kuiken C.L., Dekker J.T., Goudsmit J. (1995). Phylogeny of African monkeys based upon mitochondrial 12S rRNA sequences. J. Mol. Evol..

[40] Trifinopoulos J., Nguyen L.T., von Haeseler A., Minh B.Q. (2016). W-IQ-TREE: A fast online phylogenetic tool for maximum likelihood analysis. Nucleic Acids Res..

[41] Nesi N., Nakouné E., Cruaud C., Hassanin A. (2011). DNA Barcoding of African Fruit Bats (Mammalia; Pteropodidae). The Mitochondrial Genome Does Not Provide a Reliable Discrimination between *Epomophorus gambianus* and *Micropteropus pusillus*. C. R. Biol..

[42] Diallo M.S.K., Ayouba A., Keita A.K., Thaurignac G., Sow M.S., Kpamou C., Barry T.A., Msellati P., Etard J.F., Peeters M. (2021). Temporal evolution of the humoral antibody response after Ebola virus disease in Guinea: A 60-month observational prospective cohort study. Lancet Microbe.

[43] Schuh A.J., Amman B.R., Sealy T.S., Flietstra T.D., Guito J.C., Nichol S.T., Towner J.S. (2019). Comparative analysis of serologic cross-reactivity using convalescent sera from filovirus-experimentally infected fruit bats. Sci. Rep..

[44] Penas J.A., Miranda M.E., De Los Reyes V.C., Sucaldito M.N.L., Magpantay R.L. (2019). Risk assessment of Ebola Reston virus in humans in the Philippines. West. Pac. Surveill. Response J..

[45] Bodmer B.S., Breithaupt A., Heung M., Brunetti J.E., Henkel C., Müller-Guhl J., Rodríguez E., Wendt L., Winter S.L., Vallbracht M. (2023). In vivo characterization of the novel ebolavirus Bombali virus suggests a low pathogenic potential for humans. Emerg Microbes Infect..

[46] Laing E.D., Mendenhall I.H., Linster M., Low D.H.W., Chen Y., Yan L., Sterling S.L., Borthwick S., Neves E.S., Lim J.S.L. (2018). Serologic, Evidence of Fruit Bat Exposure to Filoviruses; Singapore; 2011–2016. Emerg. Infect. Dis..

[47] Dovih P., Laing E.D., Chen Y., Low D.H.W., Ansil B.R., Yang X., Shi Z., Broder C.C., Smith G.J.D., Linster M. (2019). Filovirus-reactive antibodies in humans and bats in Northeast India imply zoonotic spillover. PLoS Negl. Trop. Dis..

[48] Brook C.E., Ranaivoson H.C., Broder C.C., Cunningham A.A., Héraud J.M., Peel A.J., Gibson L., Wood J.L.N., Metcalf C.J., Dobson A.P. (2019). Disentangling serology to elucidate henipa- and filovirus transmission in Madagascar fruit bats. J. Anim. Ecol..

[49] Schulz J.E., Seifert S.N., Thompson J.T., Avanzato V., Sterling S.L., Yan L., Letko M.C., Matson M.J., Fischer R.J., Tremeau-Bravard A. (2020). Serological Evidence for Henipa-like and Filo-like Viruses in Trinidad Bats. J. Infect. Dis..

[50] Barr J., Boyd V., Todd S., Smith I., Prada D., O’Dea M., Jackson B., Pearce L., Adams T.E., Vanderduys E. (2022). Detection of filovirus-reactive antibodies in Australian bat species. J. Gen. Virol..

[51] Cappelle J., Furey N., Hoem T., Ou T.P., Lim T., Hul V., Heng O., Chevalier V., Dusart P., Duong V. (2021). Longitudinal monitoring in Cambodia suggests higher circulation of alpha and betacoronaviruses in juvenile and immature bats of three species. Sci. Rep..

[52] Hayman D.T.S. (2015). Biannual birth pulses allow filoviruses to persist in bat populations. Proc. R. Soc. B Biol. Sci..

[53] Reed Hranac C., Marshall J.C., Monadjem A., Hayman D.T.S. (2019). Predicting Ebola virus disease risk and the role of African bat birthing. Epidemics.

[54] Jones M.E., Schuh A.J., Amman B.R., Sealy T.K., Zaki S.R., Nichol S.T., Towner J.S. (2015). Experimental Inoculation of Egyptian Rousette Bats (*Rousettus aegyptiacus*) with Viruses of the Ebolavirus and Marburgvirus Genera. Viruses.

[55] Science, this Bat Species May Be the Source of the Ebola Epidemic That Killed more than 11,000 People in West Africa. https://www.science.org/content/article/bat-species-may-be-source-ebola-epidemic-killed-more-11000-people-west-africa.

[56] Bokelmann M., Vogel U., Debeljak F., Düx A., Riesle-Sbarbaro S., Lander A., Wahlbrink A., Kromarek N., Neil S., Couacy-Hymann E. (2021). Tolerance and Persistence of Ebola Virus in Primary Cells from *Mops condylurus*; a Potential Ebola Virus Reservoir. Viruses.

[57] Hermida Lorenzo R.J., Cadar D., Koundouno F.R., Juste J., Bialonski A., Baum H., García-Mudarra J.L., Hakamaki H., Bencsik A., Nelson E.V. (2021). Metagenomic Snapshots of Viral Components in Guinean Bats. Microorganisms.

[58] Swanepoel R., Leman P.A., Burt F.J., Zachariades N.A., Braack L.E., Ksiazek T.G., Rollin P.E., Zaki S.R., Peters C.J. (1996). Experimental inoculation of plants and animals with Ebola virus. Emerg. Infect. Dis..

[59] Peel A.J., Wood J.L.N., Baker K.S., Breed A.C., de Carvalho A., Fernández-Loras A., Gabrieli H.S., Gembu G.-C., Kakengi V.A., Kaliba P.M. (2017). How Does Africa’s Most Hunted Bat Vary Across the Continent? Population Traits of the Straw-Coloured Fruit Bat (*Eidolon helvum*) and Its Interactions with Humans. Acta Chiropterol..

[60] Kamins A., Restif O., Ntiamoa-Baidu Y., Suu-Ire R., Hayman D., Cunningham A., Wood J., Rowcliffe M. (2011). Uncovering the fruit bat bushmeat commodity chain and the true extent of fruit bat hunting in Ghana; West Africa. Biol. Conserv..

[61] Baudel H., De Nys H., Ngole E.M., Peeters M., Desclaux A. (2019). Understanding Ebola virus and other zoonotic transmission risks through human–bat contacts: Exploratory study on knowledge; attitudes and practices in Southern Cameroon. Zoonoses Public Health.

[62] Frick W.F., Kingston T., Flanders J. (2020). A review of the major threats and challenges to global bat conservation. Ann. N. Y. Acad. Sci..

[63] Euren J., Bangura J., Gbakima A., Sinah M., Yonda S., Lange C.E., McIver D.J., LeBreton M., Wolking D., Grigorescu Monagin C. (2020). Human Interactions with Bat Populations in Bombali; Sierra Leone. EcoHealth.

