# Supplementary material for: Extensive Survey and Analysis of Factors Associated with Presence of Antibodies to Orthoebolaviruses in Bats from West and Central Africa
Source: Viruses. 2023 Sep 15;15(9):1927. doi: 10.3390/v15091927 (PMC10536003; doi:10.3390/v15091927)
Supplement: Supplementary file 1 [file viruses-15-01927-s001.zip › viruses-2569389-supplementary.pdf]

Supplementary Table S1. Seroprevalence of IgG antibodies to GP SUDV (less stringent cut-off) according to sex for the different bat species in Guinea, Cameroon and DRC.

	Females			Males		
	n+/N tested	%+	(95%CI)	n+/N tested	%+	(95%CI)
**Frugivorous bats**						
**Pteropodidae**						
Eidolon helvum	361/828	43.6	(40.3-47.0)	359/818	43.9	(40.5-47.3)
Epomophorus sp.[a]	33/388	8.5	(6.1-11.7)	16/220	7.3	(4.5-11.5)
Epomops sp.[b]	9/492	1.8	(1.0-3.4)	12/568	2.1	(1.2-3.7)
Hypsignathus monstrosus	97/355	27.3	(22.9-32.2)	45/212	21.2	(16.3-27.2)
Lissonycteris angolensis	19/113	16.8	(11.0-24.8)	8/73	11	(5.7-20.2)
Micropteropus pusillus	11/136	8.1	(4.6-0-13.9)	5/119	4.2	(1.8-9.5)
Myonycteris torquata	14/186	7.5	(4.5-12.2)	14/207	6.8	(4.1-11.0)
Rousettus aegyptiacus	147/654	22.5	(19.4-25.8)	110/471	23.4	(19.8-27.4)
Other frugivorous species[c]	0/33	0	(0-10.4)	0/43	0	(0-8.2)
**Insectivorous bats**						
**Molossidae**						
Chaerephon/Mops [d]	32/815	3.9	(2.8-5.5)	18/601	3	(1.9-4.7)
**Hipposideridae**						
Hipposideros sp. [d]	4/768	0.5	(0.2-1.3)	4/799	0.5	(0.2-1.3)
**Miniopteridae**						
Miniopterus sp. [d]	3/136	2.2	(0.8-6.3)	4/125	3.2	(1.3-7.9)
**Nycteridae**						
Nycteris sp. [d]	0/62	0	(0-5.8)	0/24	0	(0-13.8)
**Rhinolophidae**						
Rhinolophus sp. [d]	0/56	0	(0-6.4)	0/89	0	(0-4.1)
Other insectivorous species[e]	0/58	0	(0-6.2)	0/39	0	(0-9)
**Total**	**730/5,080**	**14.4**	**(13.4-15.4)**	**595/4,408**	**13.5**	**(12.5-14.5)**

[a] Two *Epomophorus* species were observed, *E. gambianus* in Guinea, Cameroon and Western DRC and *E. labiatus* in eastern DRC.

[b] Two *Epomops* species were observed, *E. franqueti* in Cameroon and DRC and *E. buettikoferi* in Guinea.

[c] Other frugivorous species; *Casinycteris* sp. (n=18), *Megaloglossus woermani* (n=55), *Nanonycteris* sp. (n=3) and *Scotonycteris* sp. (n=3)

[d] Identification at species level was not possible for a significant proportion of samples tested and were therefore grouped at the genus level. In the Molossidae family, differentiation between *Chaerephon* and *Mops* species was often not possible and they were analyzed together.

[e] Other insectivorous species; *Coleura afra* (n=6), *Glauconycteris* sp.(n=8), *Kerivoula* sp. (n=1), *Myotis* sp. (n=4), *Neoromicia* sp. (n=26), *Scotophilus* sp. (n=45) and *Taphozous mauritianus* (n=7).

Supplementary Table S2. Seroprevalence of IgG antibodies to GP SUDV (less stringent cut-off) for juveniles and adults (subadults and mature adults) for the different bat species in Guinea, Cameroon and DRC.

	**Juveniles**			**Adults**		
	n+/N tested	%+	(95%CI)	n+/N tested	%+	(95%CI)
**Frugivorous bats**						
**Pteropodidae**						
Eidolon helvum	40/274	14.6	(10.9-19.3)	668/1,355	49.3	(46.6-52)
Epomophorus sp. [a]	4/31	12.9	(5.1-28.9)	45/573	7.9	(6-10.4)
Epomops sp. [b]	3/201	1.5	(0.5-4.3)	18/842	2.1	(1.4-3.4)
Hypsignathus monstrosus	22/180	12.2	(8.2-17.8)	113/372	30.4	(26-35.2)
Lissonycteris angolensis	0/13	0	(0-22.8)	24/167	14.4	(9.9-20.5)
Micropteropus pusillus	0/18	0	(0-17.6)	16/228	7	(4.4-11.1)
Myonycteris torquata	2/83	2.4	(0.7-8.4)	26/308	8.4	(5.8-12.1)
Rousettus aegyptiacus	36/182	19.8	(14.6-26.2)	212/919	23	(20.5-25.9)
Other frugivorous species [c]	0/6	0	na[f]	0/69	0	(0-5.3)
**Insectivorous bats**						
**Molossidae**						
Chaerephon/Mops [d]	4/68	5.9	(2.3-11.8)	40/1,303	3.1	(2.3-4.2)
**Hipposideridae**						
Hipposideros sp. [d]	0/70	0	(0-5.2)	7/1248	0.6	(0.3-1.2)
**Miniopteridae**						
Miniopterus sp. [d]	1/52	1.9	(0.1-10.1)	6/197	3.1	(1.4-6.5)
**Nycteridae**						
Nycteris sp. [d]	0/3	0	na	0/83	0	(0-4.4)
**Rhinolophidae**						
Rhinolophus sp. [d]	0/3	0	na	0/123	0	(0-3)
Other insectivorous species [e]	0/2	0	na	0/86	0	(0-4.3)
**Total**	**112/1,186**	**9.4**	**(7.9-11.2)**	**1,175/7,873**	**14.9**	**(14.2-15.7)**

[a] Two *Epomophorus* species were observed, *E. gambianus* in Guinea, Cameroon and Western DRC and *E. labiatus* in eastern DRC.

[b] Two *Epomops* species were observed, *E. franqueti* in Cameroon and DRC and *E. buettikoferi* in Guinea.

[c] Other frugivorous species; *Casinycteris* sp. (n=18), *Megaloglossus woermani* (n=55), *Nanonycteris* sp. (n=3) and *Scotonycteris* sp. (n=3)

[d] Identification at species level was not possible for a significant proportion of samples tested and were therefore grouped at the genus level. In the Molossidae family, differentiation between *Chaerephon* and *Mops* species was often not possible and they were analyzed together.

[e] Other insectivorous species; *Coleura afra* (n=6), *Glauconycteris* sp.(n=8), *Kerivoula* sp. (n=1), *Myotis* sp. (n=4), *Neoromicia* sp. (n=26), *Scotophilus* sp. (n=45) and *Taphozous mauritianus* (n=7).

[f] Not applied because there were no samples tested or unsufficient samples to calculate percentages and/or 95%CI

Supplementary Table S3. Seroprevalence of IgG antibodies to GP SUDV (less stringent cut-off) for juveniles, subadults and adults for the different bat species in Cameroon.

	**Juveniles**			**Subadults**			**Adults**		
	n+/N tested	%+	(95%CI)	n+/N tested	%+	(95%CI)	n+/N tested	%+	(95%CI)
**Frugivorous bats**									
**Pteropodidae**									
Eidolon helvum	40/268	14.9	(11.2-19.7)	158/226	69.9	(63.6-75.5)	286/506	56.5	(52.2-60.8)
Epomophorus sp. [a]	4/8	50	(21.5-78.5)	0/0	na[f]	na	1/8	12.5	(0.6-47.1)
Epomops sp. [b]	3/195	1.5	(0.5-4.4)	0/31	0	(0.0-11.0)	12/587	2	(1.2-3.5)
Hypsignathus monstrosus	22/180	12.2	(8.2-17.8)	5/28	17.9	(7.9-35.6)	107/314	34.1	(29.1-39.5)
Lissonycteris angolensis	0/6	0	na	0/5	0	na	20/79	25.3	(17-36)
Micropteropus pusillus	0/6	0	na	1/10	10	(0.5-40.4)	0/14	0	(0-21.5)
Myonycteris torquata	1/72	1.39	(0.07-7.5)	1/18	5.6	(0.3-25.8)	22/198	11.1	(7.5-16.2)
Rousettus aegyptiacus	32/170	19.9	(13.6-25.3)	30/75	40	(29.7-51.3)	95/300	31.7	(26.7-37.1)
Other frugivorous species [c]	0/5	0	na	0/4	0	na	0/31	0	(0-11)
**Insectivorous bats**									
**Molossidae**									
Chaerephon/Mops [d]	4/51	7.8	(3.1-18.5)	0/0	na	na	23/263	8.7	(6-12.8)
**Hipposideridae**									
Hipposideros sp. [d]	0/44	0	(0-8)	3/27	11.1	(3.9-28.1)	3/596	0.5	(0.2-1.5)
**Miniopteridae**									
Miniopterus sp. [d]	0/0	na	na	1/5	20	(1-62.5)	0/3	0	na
**Nycteridae**									
Nycteris sp. [d]	0/1	0	na	0/0	na	na	0/4	0	na
**Rhinolophidae**									
Rhinolophus sp. [d]	0/1	0	na	0/8	0	na	0/37	0	(0-9.4)
Other insectivorous species [e]	0/0	na	na	0/1	0	na	0/49	0	(0-7.3)
**Total**	**106/1,007**	**10.5**	**(8.8-12.6)**	**199/438**	**45.4**	**(40.8-50.1)**	**569/2,959**	**19.2**	**(17.8-20.7)**

[a] Two *Epomophorus* species were observed, *E. gambianus* in Guinea, Cameroon and Western DRC and *E. labiatus* in eastern DRC.

[b] Two *Epomops* species were observed, *E. franqueti* in Cameroon and DRC and *E. buettikoferi* in Guinea.

[c] Other frugivorous species; *Casinycteris* sp. (n=18), *Megaloglossus woermani* (n=55), *Nanonycteris* sp. (n=3) and *Scotonycteris* sp. (n=3)

[d] Identification at species level was not possible for a significant proportion of samples tested and were therefore grouped at the genus level. In the Molossidae family, differentiation between *Chaerephon* and *Mops* species was often not possible and they were analyzed together.

[e] Other insectivorous species; *Coleura afra* (n=6), *Glauconycteris* sp.(n=8), *Kerivoula* sp. (n=1), *Myotis* sp. (n=4), *Neoromicia* sp. (n=26), *Scotophilus* sp. (n=45) and *Taphozous mauritianus* (n=7).

[f] Not applied because there were no samples tested or unsufficient samples to calculate percentages and/or 95%CI

Supplementary Table S4: Seroprevalence against GP SUDV (less stringent cut-off) for gestating and non-gestating adult female bats for the different bat species in Guinea, Cameroon and DRC.

	Gestation			No Gestation		
	n+/N tested	%+	(95%CI)	n+/N tested	%+	(95%CI)
**Frugivorous bats**						
**Pteropodidae**						
Eidolon helvum	8/19	42.1	(23.1-63.7)	115/298	38.6	(33.2-44.2)
Epomophorus sp.[a]	2/30	6.7	(1.8-21.3)	24/298	8.1	(5.5-11.7)
Epomops sp.[b]	0/26	0	(0-12.9)	3/128	2.3	(0.8-6.7)
Hypsignathus monstrosus	4/23	17.4	(7-37.1)	38/124	30.6	(23.2-39.2)
Lissonycteris angolensis	1/11	9.1	(0.5-37.7)	7/48	14.6	(7.2-27.2)
Micropteropus sp.	4/14	28.6	(11.7-54.6)	2/34	5.9	(1.6-19.1)
Myonycteris sp.[c]	0/15	0	(0-20.4)	3/38	7.9	(2.7-20.8)
Rousettus aegyptiacus	9/48	18.8	(10.2-31.9)	70/383	18.3	(14.7-22.5)
Other frugivorous species[d]	0/4	0	na[f]	0/11	0	(0-25.9)
**Insectivorous bats**						
**Molossidae**						
Chaerephon/Mops [c]	1/56	1.8	(0-9.4)	12/603	2	(1.1-3.4)
**Hipposideridae**						
Hipposideros sp. [c]	0/81	0	(0-4.5)	2/332	0.6	(0.2-2.2)
**Miniopteridae**						
Miniopterus sp. [c]	0/11	0	(0-25.9)	0/30	0	(0-11.4)
**Nycteridae**						
Nycteris sp. [c]	0/2	0	na	0/56	0	(0-6.4)
**Rhinolophidae**						
Rhinolophus sp. [c]	0/1	0	na	0/31	0	(0-11)
Other insectivorous species[e]	0/3	0	na	0/36	0	(0-9.6)
**Total**	**29/344**	**8.4**	**(5.9-11.8)**	**276/2450**	**11.3**	**(10.1-12.6)**

[a] Two *Epomophorus* species were observed, *E. gambianus* in Guinea, Cameroon and Western DRC and *E. labiatus* in eastern DRC.

[b] Two *Epomops* species were observed, *E. franqueti* in Cameroon and DRC and *E. buettikoferi* in Guinea.

[c] Other frugivorous species; *Casinycteris* sp. (n=18), *Megaloglossus woermani* (n=55), *Nanonycteris* sp. (n=3) and *Scotonycteris* sp. (n=3)

[d] Identification at species level was not possible for a significant proportion of samples tested and were therefore grouped at the genus level. In the Molossidae family, differentiation between *Chaerephon* and *Mops* species was often not possible and they were analyzed together.

[e] Other insectivorous species; *Coleura afra* (n=6), *Glauconycteris* sp.(n=8), *Kerivoula* sp. (n=1), *Myotis* sp. (n=4), *Neoromicia* sp. (n=26), *Scotophilus* sp. (n=45) and *Taphozous mauritianus* (n=7).

[f] Not applied because there were no samples tested or unsufficient samples to calculate percentages and/or 95%CI

Supplementary Table S5: Seroprevalence against GP SUDV (less stringent cut-off) for lactating and non-lactating adult female bats for the different bat species in Guinea, Cameroon and DRC.

	**Lactation**			**No Lactation**		
	n+/N tested	%+	(95%CI)	n+/N tested	%+	(95%CI)
**Frugivorous bats**						
**Pteropodidae**						
Eidolon helvum	22/88	25	(17.1-35)	227/475	47.8	(43.3-52.3)
Epomophorus sp.[a]	4/53	7.5	(3-17.9)	25/298	8.4	(5.7-12.1)
Epomops sp.[b]	1/45	2.2	(0.1-11.6)	6/321	1.9	(0.9-4)
Hypsignathus monstrosus	12/44	27.3	(16.3-41.8)	63/174	36.2	(29.4-43.6)
Lissonycteris angolensis	1/7	14.3	(0.7-51.3)	15/92	16.3	(10.1-25.2)
Micropteropus sp.	0/5	0	na[f]	11/107	10.3	(5.8-17.5)
Myonycteris sp.[c]	1/18	5.6	(0.3-25.8)	11/122	9	(5.1-15.4)
Rousettus aegyptiacus	18/88	20.5	(13.3-30)	82/401	20.4	(16.8-24.7)
Other frugivorous species[d]	0/0	na	na	0/25	0	(0-13.3)
**Insectivorous bats**						
**Molossidae**						
Chaerephon/Mops [c]	1/23	4.3	(0.2-21)	18/700	2.6	(1.6-4)
**Hipposideridae**						
Hipposideros sp. [c]	0/30	0	(0-11.4)	3/485	0.6	(0.2-1.8)
**Miniopteridae**						
Miniopterus sp. [c]	0/0	na	na	1/60	1.7	(0.1-8.9)
**Nycteridae**						
Nycteris sp. [c]	0/3	0	na	0/55	0	(0-6.5)
**Rhinolophidae**						
Rhinolophus sp. [c]	0/3	0	na	0/35	0	(0-9.9)
Other insectivorous species[e]	0/1	0	na	0/50	0	(0-7.1)
**Total**	**60/408**	**14.7**	**(11.6-18.5)**	**462/3400**	**13.6**	**(12.5-14.8)**

[a] Two *Epomophorus* species were observed, *E. gambianus* in Guinea, Cameroon and Western DRC and *E. labiatus* in eastern DRC.

[b] Two *Epomops* species were observed, *E. franqueti* in Cameroon and DRC and *E. buettikoferi* in Guinea.

[c] Other frugivorous species; *Casinycteris* sp. (n=18), *Megaloglossus woermani* (n=55), *Nanonycteris* sp. (n=3) and *Scotonycteris* sp. (n=3)

[d] Identification at species level was not possible for a significant proportion of samples tested and were therefore grouped at the genus level. In the Molossidae family, differentiation between *Chaerephon* and *Mops* species was often not possible and they were analyzed together.

[e] Other insectivorous species; *Coleura afra* (n=6), *Glauconycteris* sp.(n=8), *Kerivoula* sp. (n=1), *Myotis* sp. (n=4), *Neoromicia* sp. (n=26), *Scotophilus* sp. (n=45) and *Taphozous mauritianus* (n=7).

[f] Not applied because there were no samples tested or unsufficient samples to calculate percentages and/or 95%CI.
